# P38 Signal Transduction Pathway Has More Cofactors on Apoptosis of SGC-7901 Gastric Cancer Cells Induced by Combination of Rutin and Oxaliplatin

**DOI:** 10.1155/2019/6407210

**Published:** 2019-11-06

**Authors:** Qi Li, Liqun Ren, Yang Zhang, Zehui Gu, Qi Tan, Tong Zhang, Meng Qin, Suxian Chen

**Affiliations:** ^1^Department of Pathology, The Third Affiliated Hospital of Jinzhou Medical University, Jinzhou, Liaoning, China; ^2^Department of Experimental Pharmacology and Toxicology, School of Pharmaceutical Sciences, Jilin University, Changchun, Jilin, China; ^3^Department of Pathology and Pathophysiology, Jinzhou Medical University, Jinzhou, Liaoning, China

## Abstract

Currently, gastric cancer treatment is mainly based on first-line intervention with oxaliplatin (OXA) after surgical resection, but the application of OXA has been limited due to the toxic side effects caused by the cumulative dose. The toxicity of OXA mainly includes hepatotoxicity, nephrotoxicity, and ototoxicity, and there is an urgent clinical need to find alternatives that are less toxic and more effective. Rutin (RT) is a natural flavonoid with many biological activities. Studies have found that RT inhibits tumor cell growth and enhances their sensitivity toward certain drugs. As the underlying impact of RT on gastric cancer and its molecular mechanism remain poorly understood, we performed a series of experiments to determine whether RT has the effect of treating gastric cancer, and whether it can cooperate with OXA to treat gastric cancer and its related mechanisms. In the present study, we founded that RT suppressed cell viability, inhibited cell proliferation by causing G0/G1 arrest, and induced apoptosis in SGC 7901 cells. And RT can play as an antitumor agent together with OXA. The mechanism of RT-induced apoptosis may be associated with the activation of the p38/Caspase signal pathway. These results demonstrated the potential of RT as a promising therapeutic compound to treat gastric cancer. At the same time, RT can synergize with OXA to reduce the dose of OXA and reduce the toxicity.

## 1. Introduction

Gastric cancer, one of the most common malignant tumors worldwide, is caused by the gastric mucosal epithelium, severely affects patients' health and quality of life. There are obvious regional differences in its occurrence, with Japan, Korea, and China being high-risk areas in Asia. In particular, gastric cancer is one of the most prominent malignant tumors in China. It accounts for the fifth highest incidence rate and third highest mortality rate worldwide, causing heavy medical burdens [1]. Existing studies have reported that the pathogenesis of gastric cancer involves changes in tumor suppressor genes, oncogenes, apoptotic genes, and transfer genes. p38 mitogen-activated protein kinase (MAPK) is the most important member of the MAPK family. It is involved in cell survival, differentiation, apoptosis, and cell cycle regulation. In recent years, the p38 MAPK pathway has been found to regulate multiple functions of tumor cells such as invasion, differentiation, metastasis, proliferation, and apoptosis [2]. It has also been found to be abnormally activated in a variety of tumors, including gastric cancer tissues [3, 4]. Therefore, a more in-depth study of the p38 MAPK signal transduction pathway may provide a new target for the treatment and prognosis of gastric cancer. In this study, RT was combined with OXA, a first-line chemotherapeutic drug for gastric cancer, to treat SGC-7901 human gastric cancer cells. The effects of RT on SGC-7901 cell proliferation and apoptosis were observed. The role of the p38 MAPK signal transduction pathway provides a theoretical and experimental basis for the clinical treatment of gastric cancer.

## 2. Materials and Methods

### 2.1. Materials

#### 2.1.1. Cell Line

The human normal gastric mucosal epithelial cell lines NGEC was acquired from the College of Basic Medicine of Jilin University, human gastric cancer cell lines SGC-7901 was acquired from the laboratory of the College of Life Sciences of Jilin University and frozen before use.

#### 2.1.2. Drugs and Reagents

The following products were used: RT (Nanjing Jingzhu Biotech Co., Ltd., Nanjing, China), OXA for injection (H20000337; Jiangsu HengRui Pharmaceutical Co., Ltd., Jiangsu, China), p38 inhibitor (SB203580; Sigma, USA), high-sugar medium (Hyclone, USA), excellent grade fetal bovine serum (Tianjin Haouyang Biological Products Technology Co., Ltd., Tianjin, China), thiazole blue dye solution (MTT, Sigma, USA), dimethyl sulfoxide (DMSO, Sigma, USA), Hoechst 33258 staining solution (Shanghai Biyuntian Biotechnology Research Institute, Shanghai, China), Annexin V-FITC/PI Apoptosis Detection Kit (Sigma, USA), rabbit antihuman monoclonal antibodies against p38, phosphorylated p38 (p-p38), B-cell lymphoma 2 (Bcl-2), Bcl-2-associated X protein (Bax), procaspase-3, cleaved caspase-3, cleaved caspase-7, cleaved caspase-8, and cleaved caspase-9, horseradish peroxidase-labeled goat antirabbit/mouse secondary antibody (Beijing Zhongshangjinqiao Biotechnology Co., Ltd., Beijing, China), Trizol (Invitrogen, USA), Tween-20, sodium dodecyl sulfate, ammonium persulfate, acrylamide, Tris-HCl, glycine (Beijing DingGuoChangSheng Co., Ltd., Beijing, China), *N*,*N*′-methylene bis acrylamide, and total protein extraction kit (Beijing Genomics Puli Lai, Beijing, China).

#### 2.1.3. Instrumentation

The following instruments were used: inverted phase-contrast microscope, biological microscope (Nikon, Japan), electrophoresis tank, flow cytometer, CO_2_ constant temperature incubator, plate reader, centrifuge, and JJ260 precision electronic balance (American BD company).

### 2.2. Methods

#### 2.2.1. Cell Culture

Cells were maintained in Dulbecco's modified Eagle medium supplemented with 10% fetal bovine serum, 100 units/mL penicillin, and 10 mg/mL streptomycin. Cells were cultured in a humidified atmosphere containing 5% CO_2_ at 37°C and digested with 0.25% trypsin once every 2-3 days.

#### 2.2.2. Drug Treatment

A stock solution of RT was prepared at 1 mol/L in DMSO and stored at −20°C. The solution was diluted with serum-free medium and the concentration of DMSO was controlled below 0.1%. The experimental set-up was as follows: the human normal gastric mucosal epithelial cells NGEC and gastric cancer cells SGC-7901 were treated with different concentrations of RT and OXA; then, the following grouping was conducted with SGC-7901 as the research object: (1) negative control (no drug administration), (2) p38 inhibitor (SB203580, 15 nM), (3) RT (300 *μ*M), (4) OXA (33 *μ*M), (5) RT (300 *μ*M) and OXA (33 *μ*M), (6) RT (300 *μ*M) and SB203580 (15 nM), (7) OXA (33 *μ*M) and SB203580 (15 nM), and (8) combination of OXA (33 *μ*M) and RT (300 *μ*M) and SB203580 (15 nM). After 24 h of treatment, changes in cell morphology and density were observed. The medium-effect principle was used to calculate the drug concentration and the combined dose. The inhibition rate (*f*_a_) = 1—(average optical density of the experimental group/average optical density of the control group) according to the medium-effect equation *f*_a_/*f*_u_ = (*D*/*D*_m_). The intermediate concentration Dm of each drug alone and in combination, which is the basis for the above drug concentration grouping and the combined dose, was calculated. The combined index (CI) = *D*_1_/*D*_*x*1_ + *D*_2_/*D*_*x*2_ + *αD*_1_*D*_2_/*D*_*x*1_*D*_*x*2_ for various effects when the two drugs are combined, CI < 1, is calculated as a synergy. Apoptosis was observed after 24 h of drug action when the morphology of apoptotic cells became clearly visible.

### 2.3. Biological Characterization

#### 2.3.1. Determination of Cell Viability by 3-(4,5-dimethylthiazol-2-yl)-2,5-diphenyltetrazolium Bromide (MTT) Assay

The effect of the drugs on cell viability was examined by the MTT assay. Briefly, cells were seeded into 96-well plates (5 × 10^4^ cells per well) and treated with the indicated drugs. After 24 h of incubation, 20 *μ*L of MTT (5 mg/mL) solution was added to each well. The absorbance was measured using a 96-well plate reader at a wavelength of 490 nm after 4 h of incubation. The experiment was repeated three times. Cell survival rate = [experimental group *D* (490)/control group *D* (490)] × 100%.

#### 2.3.2. Hoechst 33258 Fluorescent Staining

A suspension of SGC-7901 cells was prepared at 1 × 10^6^ cells/mL and inoculated at 1 mL per well into a 6-well plate on sterilized coverslips, in triplicates for each group. After 24 h of dosing, the cells were collected and washed three times with phosphate-buffered saline (PBS). Then, the cells were fixed with 0.5 mL of 10% formaldehyde at 4°C for 15 min, after which the fixative was removed and the cells were washed three times with PBS. The cells were stained with 0.5 mL of Hoechst 33258 solution for 3 min at 25°C and sealed with antifluorescence film. The morphological changes in apoptotic cells were observed directly under a fluorescence microscope after the film was sealed, and images were acquired using the Image Advanced 3.2 system.

#### 2.3.3. Flow Cytometry for Cell Cycle Detection

A suspension of SGC-7901 cells in the logarithmic growth phase was prepared at 1 × 10^6^ cells/mL. The cells were seeded in a 6-well plate at 1 mL per well. After drug administration, the cells were collected and centrifuged at 1000 rpm for 5 min. The supernatant was discarded and the cells were washed with 1 mL of PBS. Precooled 70% ethanol solution was added at 4°C for 12 h and discarded by centrifugation, after which the cells were washed twice with PBS. The PBS was discarded, and 1 mL of propidium iodide (PI) solution was added at 37°C. The cells were resuspended after incubation for 30 min in the dark and subjected to flow cytometry within 30 min. The experiment was repeated three times.

#### 2.3.4. Flow Cytometry for Apoptosis Detection

After the above operation, the cells were collected, centrifuged at 4°C, and washed twice with precooled PBS. Binding buffer (200 *μ*L) was added to resuspend the cells and 10 *μ*L of Annexin V-fluorescein isothiocyanate was added. The cells were incubated in the absence of light for 15 min. Next, 300 *μ*L of binding buffer and 10 *μ*L of PI were added, and the cells were subjected to flow cytometry within 1 h. The experiment was repeated three times.

#### 2.3.5. Western Blot of Related Proteins

A suspension of SGC-7901 cells in logarithmic growth phase was prepared at 1 × 10^6^ cells/mL. After 24 h of drug administration, total proteins were extracted according to the instructions of the total protein extraction kit. The protein concentration was detected by the bicinchoninic acid method, and 10 *μ*L of protein was loaded into each well for sodium dodecyl sulfate-polyacrylamide gel electrophoresis. The proteins were transferred to polyvinylidene fluoride membranes and blocked for 2 h. Thereafter, the membranes were incubated at 4°C overnight with primary antibodies against p38 (1 : 400), p-p38 (1 : 400), Bcl-2 (1 : 400), Bax (1 : 400), procaspase-3 (1 : 400), cleaved caspase-3 (1 : 400), cleaved caspase-7 (1 : 400), cleaved caspase-8 (1 : 400), and cleaved caspase-9 (1 : 400). The membranes were then incubated with secondary antibodies (1 : 5000) for 2 h at room temperature and treated with luminescent working solution in the dark according to the instructions of the high-sensitivity chemiluminescence detection kit. The film was exposed in a cassette, developed, and fixed. Images were acquired, and grayscale analysis was performed using Image-Pro Plus 6.0 software.

#### 2.3.6. Statistical Analysis

All reported results are representative of three independent experiments, and the data were expressed as the mean ± standard deviation. For statistical analysis, one-way analysis of variance followed by Tukey's test was performed using GraphPad Prism (San Diego, CA, USA). Differences were considered significant at *p* < 0.05, *p* < 0.01 and *p* < 0.001.

## 3. Results

### 3.1. Effect of Different Drugs on the Toxicity of NGEC Cells and the Viability of SGC-7901 Cells

**Figure 1:**
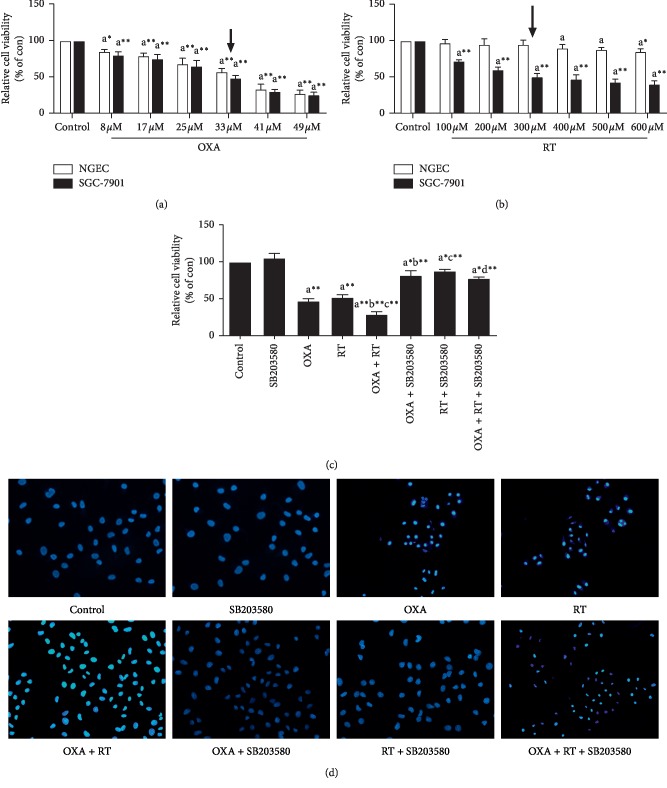
Effects of different drugs on cells. ^a^*p* < 0.05,^ a^*∗*^^*p* < 0.01,^ a^*∗∗*^^*p* < 0.001, compared with control group; ^b^*p* < 0.05,^ b^*∗*^^*p* < 0.01,^ b^*∗∗*^^*p* < 0.001, compared with OXA group; ^c^*p* < 0.05,^ c^*∗*^^*p* < 0.01,^ c^*∗∗*^^*p* < 0.001, compared with RT group; ^d^*p* < 0.05,^ d^*∗*^^*p* < 0.01,^ d^*∗∗*^^*p* < 0.001, compared with combination of OXA and RT. Control: SGC-7901/NGEC; SB203580 : 15 nM; RT: 300 *μ*M; OXA:33 *μ*M; RT + OXA: (300 *μ*M + 33 *μ*M); RT + SB203580 (300 *μ*M + 15 nM); RT + OXA + SB203580: (300 *μ*M + 33 *μ*M+15 nM). (a, b) Effect of different concentrations of RT and OXA on the toxicity of NGEC cells and the viability of SGC-7901 cells by MTT ($\bar{X}\pm S$, *n* = 6). (c) Effects of p38 inhibitor, RT, OXA, and drug combination on SGC-7901 cells proliferation assessed by MTT ($\bar{X}$ ± *S*, *n* = 6). (d) Morphological observation of SGC-7901 cells (Hoechst33258, ×200).

The results of the MTT assay showed that the viability of SGC-7901 cells treated with different concentrations of DOX and RT for 24 h was significantly inhibited compared with control group (Figures 1(a) and 1(b)), and the combined effect of the two drugs was stronger than their individual effects, while the addition of p38 inhibitor reduced the inhibitory effect of RT and OXA on cell proliferation (Figure 1(c)). In addition, small doses of RT have no obvious toxicity to human normal gastric mucosal epithelial cells, while OXA has a greater toxicity (Figures 1(a) and 1(b)).

### 3.2. Morphological Observation of Apoptosis

After Hoechst 33258 staining, the nuclei of normal SGC-7901 cells were diffusely shown in blue, whereas the nuclei of apoptotic cells were densely stained or fragmented and bright in color. Apoptosis was observed after cells were treated with RT, OXA, and a combination of the two drugs. Apoptosis was especially prominent after combination treatment, while the control cells and those treated with the p38 inhibitor showed normal nuclear morphology (Figure 1(d)).

### 3.3. Effect of Drugs on SGC-7901 Cell Cycle Progression

**Figure 2:**
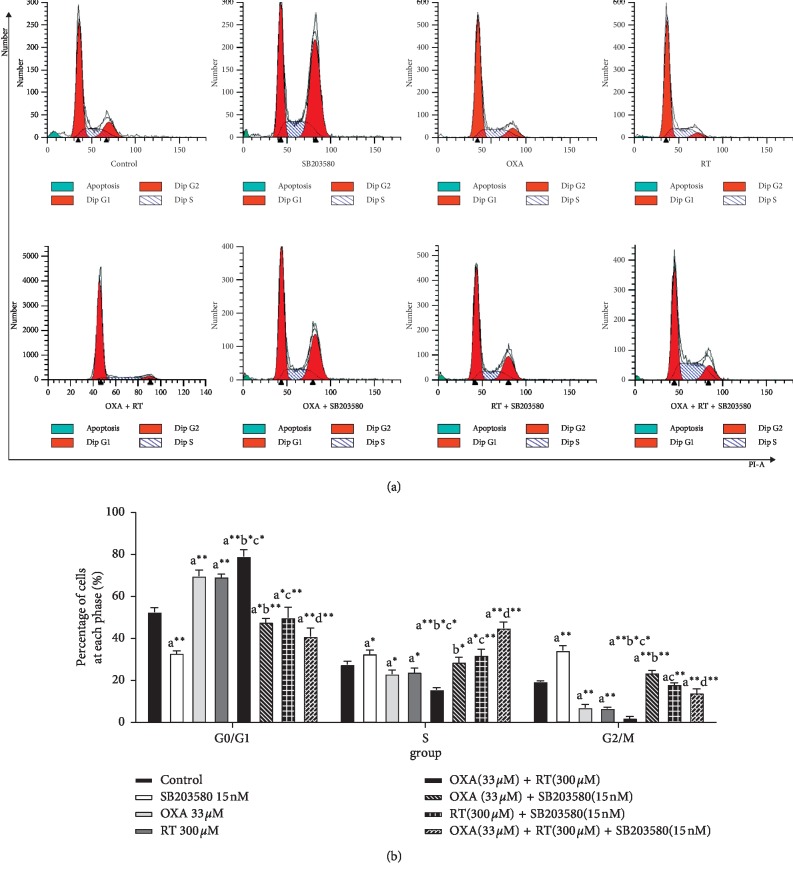
Effect of different drugs on gastric cancer cell cycle assessed by FCM ($\bar{X}\pm S$, *n* = 3). ^a^*p* < 0.05,^ a^*∗*^^*p* < 0.01,^ a^*∗∗*^^*p* < 0.001, compared with control group; ^b^*p* < 0.05,^ b^*∗*^^*p* < 0.01,^ b^*∗∗*^^*p* < 0.001, compared with OXA group; ^c^*p* < 0.05,^ c^*∗*^^*p* < 0.01,^ c^*∗∗*^^*p* < 0.001, compared with RT group; ^d^*p* < 0.05,^ d^*∗*^^*p* < 0.01,^ d^*∗∗*^^*p* < 0.001, compared with combination of OXA and RT.

After SGC-7901 cells were treated with each drug or drug combination for 24 h, they were subjected to cell cycle progression analysis. Compared with the control cells, with RT and OXA, the percentages of cells in the G0/G1 phase increased to varying degrees, and the combination treatment of RT and OXA induced the most significant change. Those after treated with p38 inhibitor showed a decrease in the percentage of cells in the G0/G1 phase. These results indicated that the RT and OXA treatment blocked cell progression in the G0/G1 phase and induced apoptosis, while the addition of p38 inhibitors inhibited the apoptosis induced by RT and OXA by accelerating the process of G0/G1 phase (Figure 2).

### 3.4. Effect of Drugs on SGC-7901 Cell Apoptosis

**Figure 3:**
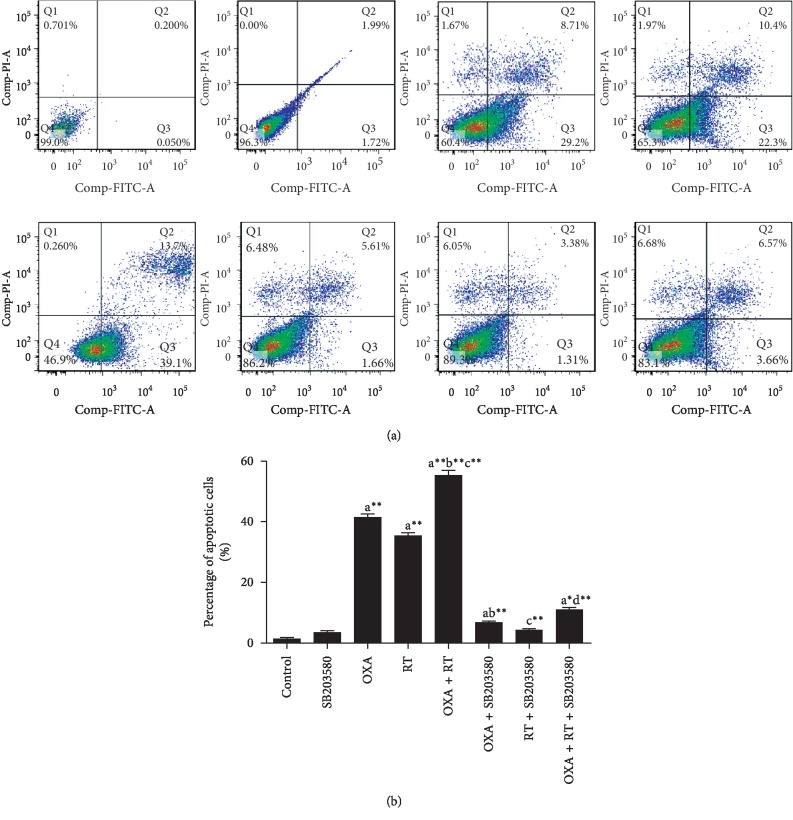
Effect of different drugs on gastric cancer cell apoptosis assessed by FCM ($\bar{X}\pm S$, *n* = 3). ^a^*p* < 0.05,^ a^*∗*^^*p* < 0.01,^ a^*∗∗*^^*p* < 0.001, compared with control group; ^b^*p* < 0.05,^ b^*∗*^^*p* < 0.01,^ b^*∗∗*^^*p* < 0.001, compared with OXA group; ^c^*p* < 0.05,^ c^*∗*^^*p* < 0.01,^ c^*∗∗*^^*p* < 0.001, compared with RT group; ^d^*p* < 0.05,^ d^*∗*^^*p* < 0.01,^ d^*∗∗*^^*p* < 0.001, compared with combination of OXA and RT.

Apoptosis was detected after cells were subjected to p38 inhibitor, RT, OXA, or combination treatment for 24 h. The early and late apoptotic rates (Q2 + Q3) were lower after p38 inhibitor treatment, with no significant difference compared with that of the control cells. The early and late apoptotic rates after RT, OXA, and combination treatment increased compared with that of the control cells, and the combination treatment induced the most significant change, while the early and late apoptotic rates were significantly reduced after the addition of p38 inhibitor (Figure 3).

### 3.5. Expression of Apoptosis-Associated Proteins

**Figure 4:**
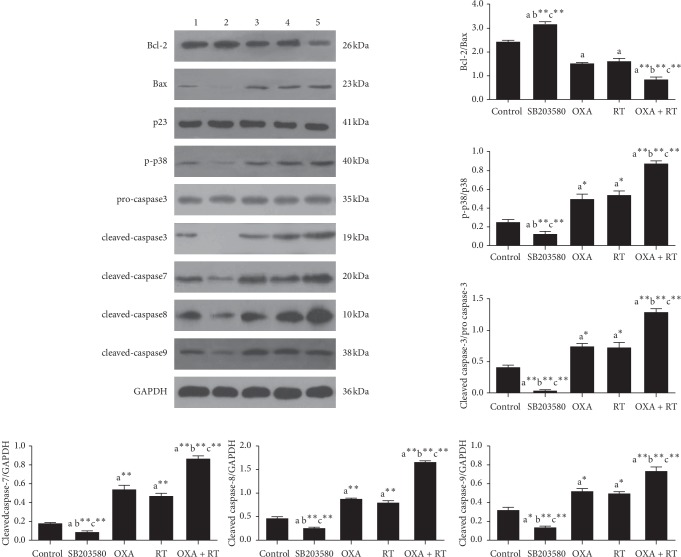
Effect of different drugs on gastric cancer cell proteins expressions assessed by western blot. 1, control group; 2, SB203580 15 nM; 3, RT 300 *μ*M; 4, OXA 33 *μ*M; 5, RT + OXA (300 *μ*M + 33 *μ*M); ^a^*p* < 0.05,^ a^*∗*^^*p* < 0.01,^ b^*∗∗*^^*p* < 0.001, compared with control group; ^b^*p* < 0.05,^ b^*∗*^^*p* < 0.01,^ b^*∗∗*^^*p* < 0.001, compared with OXA group; ^c^*p* < 0.05,^ c^*∗*^^*p* < 0.01,^ c^*∗∗*^^*p* < 0.001, compared with RT group; ^d^*p* < 0.05,^ d^*∗*^^*p* < 0.01,^ d^*∗∗*^^*p* < 0.001, compared with combination of OXA and RT.

Western blot showed that the p38 inhibitor significantly downregulated the expression of p-p38, cleaved caspase-3, cleaved caspase-7, cleaved caspase-8, cleaved caspase-9, and upregulated that of Bcl-2/Bax ratio. When RT and OXA were administered alone, the expression levels of p-p38, cleaved caspase-3, cleaved caspase-7, cleaved caspase-8, and cleaved caspase-9 were upregulated and that of Bcl-2/Bax ratio was downregulated. The effects of the combination treatment of RT and OXA were more pronounced than those of RT and OXA alone. This suggests that RT and OXA play a role in inducing apoptosis through the p38 signaling pathway (Figure 4).

## 4. Discussion

Common antitumor drugs such as OXA are reliable for the clinical treatment of gastric cancer, but long-term use of OXA can result in nephrotoxicity, ototoxicity, neurotoxicity, myelosuppression, nausea, and vomiting. Resistance of tumor cells to OXA reduces the efficacy of the drug and is a major cause of chemotherapy failure [5]. In recent years, research has increasingly focused on the synergistic effect of traditional chemotherapeutic drugs and high-efficiency Chinese medical ingredients with low toxicity. This combination ensures that while the dose of chemotherapeutic drugs is reduced, it is still sufficiently effective to kill tumor cells. Meanwhile, the toxic and side effects of the drugs are reduced and drug resistance is delayed [6]. Natural plant extracts and their flavonoids have exhibited anticancer properties in vivo and in vitro [7]. RT, a flavonoid that is widely found in many natural plants such as glutinous rice, exhibits many biological effects such as free radical scavenging, antiviral, antibacterial, and anti-inflammatory properties, gastric mucosa protection, and lowering of blood sugar [8]. RT has been used to treat prostate cancer [9], colon cancer [10], liver cancer [11], renal cell carcinoma [12], lung cancer [13], melanoma [14], and neuroblastoma [15] at the cellular level, with an inhibitory effect on tumor cell proliferation. In this study, SGC-7901 human gastric cancer cells were cultured in vitro and treated with different doses of RT. RT had a significant inhibitory effect on the growth of SGC-7901 cells, with the maximum inhibition exerted at a RT concentration of 300 *μ*M and a treatment time of 24 h, as demonstrated by the MTT assay. OXA showed a similar effect at a concentration of 33 *μ*M. The combination of the two drugs further enhanced the inhibition of SGC-7901 cell proliferation, indicating the synergistic inhibitory effect of RT and OXA on human gastric cancer cell proliferation. Iriti et al. [16] found that RT induced the apoptosis of breast cancer MDA-MB-231 cells by blocking the G0/G1 and G2/M phase. We demonstrated by flow cytometry that RT induced apoptosis in SGC-7901 cells by blocking the G0/G1 phase. RT was also shown to alleviate OXA-induced chronic neuropathic pain through its antioxidation effect [9]. Moreover, it had a certain radiation-sensitizing effect as well as a synergistic impact on tumor radiotherapy, which increased its curative effect [17].

MAPK is a serine/threonine protein kinase widely present in cells. Before activation, MAPK is located in the cytoplasm, and once activated, it enters the nucleus to activate target genes [18]. P38 is an important pathway in the MAPK family involved in apoptosis that can be activated by ultraviolet light, osmotic pressure, cytokines, antitumor drugs, physiological stress, lipopolysaccharides, and G+ bacterial cell wall components. Phosphorylated p38 enters the nucleus and subsequently regulates transcription factors, affects gene transcription, protein synthesis, and cytoskeletal structural changes, and mediates cell growth, differentiation, proliferation, and apoptosis [19]. Numerous studies have shown that p38 MAPK is involved in the process of apoptosis. Dong et al. [20] and others found that the expression of p-p38 in colorectal cancer was significantly higher than that in adenoma, and its expression in adenoma was significantly higher than that in normal colorectal mucosa. Li et al. [21] and others reported that the apoptosis of MDA-MB-231 human breast cancer cells was induced by the p38 MAPK pathway. Tang et al. [22] revealed that epigallocatechin gallate (EGCG) activated p38 and induced apoptosis of MGC803 human gastric cancer cells. After intervention with SB203580, a specific inhibitor of p38, the inhibitory effect of EGCG on the growth of MGC803 cells was significantly weakened, resulting in decreased apoptosis rate and p38 activity. Zhou et al. [23] found that toosendanin induced apoptosis of AGS and hgc-27 gastric cancer cells by blocking the G1/S phase, which may be associated with the activation of the p38 MAPK pathway. In this study, p38 phosphorylation was used as an indicator of p38 activity. RT induced p38 MAPK activation, and the consequent effect on the growth inhibition and apoptosis of SGC-7901 cells was demonstrated. After the addition of SB203580, the activity of p-p38 was inhibited, which alleviated the RT-induced growth inhibition and apoptosis. These results indicated that p38 MAPK promoted SGC-7901 cell apoptosis.

The caspase family is a protease system that directly leads to the ablation of apoptotic cells and is central to the network of apoptotic mechanisms [24]. Currently, 14 kinds of caspase have been identified and classified. According to the different positions and functions of caspases, the first class is related to apoptotic motility. These caspases are located upstream of the cascade reaction and include caspases 2, 8, 9, and 10, which can self-activate and activate downstream caspases with the participation of other proteins. The second type involves apoptotic effectors, which are located downstream of the cascade reaction. These include caspase 3, 6, and 7, which can be activated by upstream mobilization. The activated caspases act on specific substrates to exert their effects on cells, causing biochemical and morphological changes that lead to apoptosis. Class 3 includes caspases 1, 4, 5, 13, and 14, which are mainly involved in cytokine-mediated inflammatory responses and play a supporting role in death receptor-mediated apoptotic pathways. Caspase-3 is the core effector responsible for the digestion of all or parts of the key proteins in the final stage of the apoptotic pathway [25]. At the central part of the process, when cells are stimulated by apoptotic signals, the inactive precursor procaspase-3 is activated and cleaved to form an active fragment, cleaved caspase-3. This in turn activates the downstream caspase protease family and ultimately induces cell apoptosis [26]. Western blot showed that compared with the control cells, those treated with p38 inhibitor had significantly downregulated protein expression levels of cleaved caspase-3, cleaved caspase-7, cleaved caspase-8, and cleaved caspase-9. By contrast, RT and OXA treatment up-regulated the expression of these proteins. Caspase-8 and 9 are part of the first class of apoptotic initiation factors, whereas the apoptotic effectors caspase-3 and 7 belong to the second class [27]. By activating cleaved caspase-8 and cleaved caspase-9 upstream of the cascade reaction, the protein expression levels of cleaved caspase-3 and cleaved caspase-7 are increased to induce apoptosis. The activated caspase-3 further cleaves different substrates, leading to amplification of the protease cascade that eventually causes cell death. The combined effect of RT and OXA is more remarkable than those of the drugs administered alone. In addition, Bcl-2 gene encodes an integral outer mitochondrial membrane protein that blocks the apoptotic death of some cells such as lymphocytes, while Bax is the most widely studied proapoptotic protein in the Bcl-2 family. Bax protein encoded by this gene belongs to the Bcl-2 protein family. Bcl-2 family members form hetero- or homodimers and act as anti- or proapoptotic regulators that are involved in a wide variety of cellular activities. This protein forms a heterodimer with Bcl-2 and functions as an apoptotic activator. The Bcl-2/Bax ratio determines whether cells enter the apoptotic state. If Bax is dominant, then Bcl-2 is inhibited and apoptosis is induced. Otherwise, Bax is inhibited and cells survive. The results of this study showed that compared with the control group, RT and OXA decreased the Bcl-2/Bax ratio to varying degrees, and the mechanism of apoptosis may be related to activation of caspase-mediated signal transduction. The p38 inhibitor significantly upregulated the ratio of Bcl-2/Bax, further demonstrating that the p38 signaling pathway may be involved in regulating the apoptosis of SGC-7901 cells via RT and OXA.

In summary, RT inhibited the proliferation of gastric cancer cells in vitro and exhibited certain antigastric cancer activities. The combination of RT with OXA exerted a synergistic anticancer effect, downregulated the Bcl-2/Bax ratio by activating the p38 signaling pathway, and upregulated the expression of apoptotic proteins in the caspase family to trigger apoptosis in gastric cancer cells. The addition of a p38 inhibitor inhibited the expression of apoptosis-related proteins, indicating that p38 MAPK promoted the apoptosis of SGC-7901 cells. However, we did not investigate the expression of upstream substrates of the p38 signaling pathway, such as megakaryoblastic leukemia 1, apoptosis signal-regulating kinase 1, DLK, and mitogen-activated protein kinase kinase 3 and 6, and downstream substrates, such as activating transcription factor 2, CCAAT/enhancer-binding protein homologous protein 10, and myocyte-specific enhancer factor 2C. The resistance of RT in vivo was also not explored. The effect of cancer activity on gastric cancer cell invasion and metastasis and the regulation of the p38 signal transduction pathway in antigastric cancer treatment are issues that require further examination.

## Data Availability

The data used to support the findings of this study are included within the article.
